# Fear of cancer recurrence among young adult cancer survivors—exploring long-term contributing factors in a large, population-based cohort

**DOI:** 10.1007/s11764-020-00943-2

**Published:** 2020-09-28

**Authors:** Kathrine F. Vandraas, Kristin V. Reinertsen, Cecilie E. Kiserud, Hanne C. Lie

**Affiliations:** 1 National Advisory Unit for Late Effects After Cancer Treatment, Department of Oncology, Oslo University Hospital, Radiumhospitalet, Oslo, Norway; 2 Department of Behavioral Sciences in Medicine, Institute of Basic Medical Sciences, Faculty of Medicine, University of Oslo, Oslo, Norway

**Keywords:** Fear of cancer recurrence, YACSs, Long-term survivors, Post-traumatic stress

## Abstract

**Purpose:**

Fear of cancer recurrence (FCR) may be debilitating, yet knowledge of FCR among the growing population of long-term young adult cancer survivors (YACS) is scarce. We explored risk of FCR and associated factors in a nation-wide, population-based cohort of YACS.

**Methods:**

All 5-year survivors diagnosed at the ages of 19–39 years with breast cancer (BC), malignant melanoma (MM), colorectal cancer (CRC), leukemia (LEU), or non-Hodgkin lymphoma (NHL) between 1985 and 2009 in Norway were identified by the Cancer Registry of Norway and completed the cross-sectional comprehensive NOR-CAYACS health survey. Univariate and multivariate linear regression modeling was performed.

**Results:**

In total, 936 survivors were included, with an average of 16 years since diagnoses. BC was the most prevalent cancer form (38.4%), followed by MM (24.7%), NHL (15.6%), CRC (11.8%), and LEU (9.6%). Survivors worried most about getting another cancer (74%), and (20%) reported quite a bit or a lot of FCR. BC and MM survivors had the highest FCR scores. Post-traumatic stress symptoms (PTSS) had the strongest association with FCR (Std B 0.21, *p* < 0.01), above demographic and clinical variables.

**Conclusions:**

FCR is prevalent even among long-term YACS, including survivors of MM with favorable prognoses.

**Implications for Cancer Survivors:**

Attention to ongoing risks of PTSS and FCR in this growing survivor population is warranted to optimize future survivorship care.

## Background

Due to advances in diagnostics and treatment, more than 80% of young adult (YA) patients diagnosed with cancer will become long-term survivors [1]. Research on the YA cancer group is only recently gaining momentum but suggests that survivors have a high risk of late effects [2] negatively affecting their quality of life [3], and reduced life expectancy, compared with healthy controls [4]. Among adult cancer survivors, fear of cancer recurrence (FCR), defined as the fear, worry, or concern relating to the possibility that cancer will come back or progress [5], is increasingly recognized as one of the most prevalent and stressful symptoms [6]. The only systematic review on FCR among adolescent and young adult (AYA) cancer survivors reported highly varying prevalence estimates ranging from 31 to 82.5% [7]. In this population, FCR was associated with increased risk of impaired physical and psychological functioning, and reduced over-all quality of life. These findings, combined with high rates of reported unmet supportive and psychological follow-up needs in the same population [8], warrant further investigation of FCR and contributing factors to optimize long-term outcomes for survivors of YA cancers [8, 9].

Young adulthood, defined as the age period from 19 to 39 years [10], is a critical transitional phase where attainment of life-milestones, such as completion of education and forming of a career, relationships, and a family, is in focus [11]. A cancer diagnosis may derail this developmental trajectory. Being confronted with a potentially deadly disease at a young age and subsequently surviving it may result in post-traumatic stress symptoms (PTSS) which include subclinical levels of symptoms of post-traumatic stress disorder (PTSD), characterized by intrusive thoughts and avoidant behavior [12]. In younger age groups, PTSS has predominately been demonstrated in studies of adult survivors of childhood cancer [13]. Knowledge of PTSS among YA cancer survivors is limited, but prevalence rates of 39–50% have been reported in early phases of survivorship (< 24 months post-diagnosis) [14]. Interestingly, the prevalence was stable at 12 months, and diagnoses with a high curation rate (90–100%) were positively associated with PTSS [15], suggesting that PTSS may persist over time, and develop irrespective of prognosis.

FCR at a low level is expected and reasonable given the severity of a cancer diagnosis, and may be beneficial for keeping survivors alert and aware of potential signs of relapse [16]. As recently as January 2020, consensus was reached for what constitutes *clinically significant* FCR, and includes the following key characteristics: high levels of preoccupation with and hypervigilance to bodily sensations or physical symptoms for signs of recurrence, and persistent worry, fear, and anxiety related to cancer recurrence [17]. In addition to hypervigilance to bodily symptoms, FCR at high levels may result in other dysfunctional behavioral patterns, including avoidance of specific situations and excessive self-checking behavior, which overlap with typical symptoms of PTSS [18]. Several studies have reported associations between PTSS and FCR, across different cancer diagnoses and disease severities [19, 20], but such knowledge is lacking in younger age groups. In the abovementioned review by Yang et al., none of the included 19 studies specifically addressed relationships between PTSS and FCR. However, the authors concluded that FCR risk seemed more dependent upon subjective psychological distress than clinical and demographical factors [7]. In a study by Skaali et al., 31% of long-term testicular cancer survivors reported quite a bit or very much FCR 11 years post-diagnosis, a finding which was clearly associated with the level of traumatic cancer-related symptoms [21].

Among adult cancer survivors, the presence of physical late effects has been reported to increase FCR risk [6]. The same association has been reported among childhood cancer survivors suffering from pain and fatigue [22]. Symptoms such as these may result in FCR through different pathways, being misinterpreted as potential signs of relapse and/or serving as constant reminders of their cancer diagnosis, interrupting emotional well-being [23].

In sum, YAs are increasingly recognized as a unique patient group in regard to cancer survivorship, with age-specific challenges and follow-up needs [11]. There are substantial knowledge gaps concerning the long-term prevalence of FCR and associated factors in this age group and within specific diagnostic groups. In order to optimize cancer care and develop age-sensitive interventions, we need to know which patients are at risk of developing FCR. The aim of this study was to explore the risk of FCR and associated factors in a population-based sample of long-term YA cancer survivors.

## Methods

This is a sub-study of the NOR-CAYACS study [24], a cross-sectional, population-based questionnaire study including all 5-year survivors of childhood, adolescent, and selected diagnostic groups of young adult cancers in Norway identified through the Norwegian Cancer Registry (CRN). The NOR-CAYACS study consists of 3558 young adults diagnosed at the ages of 19 to 39 years, with breast cancer (BC) (stage ≤ III), colon-rectal cancer (CRC), non-Hodgkin lymphoma (NHL), leukemia (LEU), or malignant melanoma (MM) between 1985 and 2009. Other relevant diagnoses were not included due to participation in concurrent research projects at our unit. Patients were included during September 2015–January 2016. Eligible participants were mailed an informed consent form and a questionnaire consisting of 302 items. One reminder was sent. In the present study, we included survivors of one cancer diagnosis only, without distant metastasis or cancer recurrence at the time of survey, not currently receiving active cancer treatment. Survivors were only included if they responded to the fear of recurrence measure and provided treatment information in the questionnaire.

The NOR-CAYACS study was approved by the Regional Committee for Medical Research Ethics (2015/232), the Norwegian Data Protection Authority (15/00395-2/CGN), the CRN, and the Data Protection Officer at Oslo University Hospital. All participants granted us with written consent upon inclusion.

FCR was assessed using the three items specifically targeting cancer worry in the Assessment of Survivor Concern (ASC) instrument [25], asking participants to rate to what extent they worry about future medical tests, cancer recurrence, and a new cancer diagnosis. Response alternatives ranged from 1 to 4, where 1 refers to not at all, 2 a little, 3 quite a bit, and 4 a lot, and where “worry” was defined as scores ≥ 2 on that item. Furthermore, responses on the three items were summed (range 3–12) with higher scores representing higher levels of FCR. Cronbach’s alpha for the FCR measure was 0.82. For the multivariate analyses, FCR was used as a continuous variable.

Information on gender, cancer type, age at diagnosis, and age at survey was obtained from the CRN, which is based on mandatory reporting and near-to-complete for the entire population [26]. All other information was self-reported. Missing data on individual items of the validated questionnaires were handled as recommended for the respective questionnaires. Survivors with incomplete responses or missing items, and where substitution or imputations were not an option, were excluded.

Educational status was dichotomized into higher education or not (> 13 or ≤ 13 years). Two questions regarding living arrangements were dichotomized as living alone or not, and with or without children. Co-morbid somatic conditions were assessed through an adapted version of Charlson co-morbidity index [27] using 13 questions on chronic diseases in major organ systems (coronary, pulmonary, gastrointestinal, kidney, neurological, rheumatic, and musculoskeletal disorders). Affirmative responses were categorized into three: no co-morbidity, 1–2, or > 2 conditions.

Information concerning cancer treatment was categorized in four groups according to treatment intensity: (1) limited surgery (including survivors of MM), (2) surgery and/or radiotherapy, (3) systemic treatment alone (chemotherapy, endocrine treatment, antibody treatment, and/or high dose chemotherapy with stem cell support/bone marrow transplantation), and (4) systemic treatment with surgery and/or radiotherapy [28]. Presence of pain was assessed using one item of the 12-item Short Form Survey (SF-12) [29], asking participants whether pain had interfered with their work-ability during the last 4 weeks. Responses were dichotomized into yes (quite a bit/extremely) or no (not at all/a little bit/moderately). Sleep was assessed using three items from the Nord-Trøndelag Health Study (The HUNT study) asking responders how often during the last 3 months they had experienced (1) difficulties falling asleep at night, (2) waking up repeatedly during the night, and (3) waking up too early without being able to go back to sleep [30]. Trouble sleeping was defined as positive responses (sometimes/many times per week) on one or more items.

Self-reported late effects included a list of 17 of the most prevalent late effects after cancer, among them coronary disease, infertility, sexual dysfunction, and cognitive deficits, on which a sum score was calculated ranging from 0 to 17. Fatigue was assessed using the Chalder’s Fatigue Questionnaire (FQ) [31], an 11-item instrument with total scores ranging from 0 to 33. Cronbach’s alpha for the inter-item correlation of the FQ was 0.92.

Anxiety was assessed using the anxiety subscale of the Hospital Anxiety and Depression Scale (HADS-A) and depression with the Patient Health Questionnaire-9 (PHQ-9) [32, 33]. The HADS-A includes seven items with four response alternatives, from 0 (not at all) to 3 (most of the time), with a range from 0 to 21. Cronbach’s alpha for HADS-A was 0.82. The PHQ-9 corresponds to the nine DSM-IV diagnostic criteria for depressive disorders and measures their severity over the past 2 weeks rated on a numeric four-point scale from 0 (not at all) to 3 (nearly every day). The sum score ranges from 0 to 27, with a higher score indicating higher symptom severity. Cronbach’s alpha for the PHQ-9 was 0.87.

The Impact of Event Scale-6 (IES-6) is a brief instrument used to measure PTSS. Six items describe symptoms of intrusion, avoidance, and hyperarousal during the last 7 days, scored from 0 to 4 according to symptom intensity (from not at all to extremely) [34]. Cronbach’s alpha for IES was 0.87.

Descriptive statistics were provided for the total sample and categorized according to diagnostic group. Group comparisons were performed using ANOVA. Univariate linear regression analyses were performed with FCR as dependent variable. Variables that were associated with FCR at a significance level set at *p* value ≤ 0.1 or stronger were then incorporated in an hierarchical, multivariate, linear model consisting of the following six blocks: block 1 (sociodemographic variables): gender, age at diagnosis, living with children or not; block 2 (clinical variables): time since diagnosis, diagnostic group, and treatment; block 3 (health variables): trouble sleeping, fatigue, and pain; block 4 (late effects): self-reported late effects; block 5 (psychological distress): anxiety; and block 6: PTSS. In block 2, MM was used as reference category for diagnostic group, and limited treatment as reference group for treatment category. In block 5, depression was not included due to the risk of inter-collinearity with anxiety. All analyses were performed using IBM SPSS statistics version 21.0 (SPSS, Chicago, IL).

## Results

Of the 3558 survivors, 1488 (42%) consented and completed the questionnaire. For this sub-study, 936 survivors met the inclusion criteria.

The survivors were primarily female (72.9%), with higher education (58.4%) and cohabitating (82.2%) at the time of survey. Almost half of the sample lived with children (47.6%). Close to 16 years (15.9 years, SD 6.6 years) had passed since their cancer diagnosis. At diagnosis, survivors of LEU were the youngest (28.8 years), while survivors of BC were the oldest (35.2 years). BC was the largest diagnostic group in the sample (38.4%). Close to all survivors of MM had been treated with limited surgery only (93.5%), but the majority of the total sample had received systemic therapy with surgery and/or radiotherapy (54.1%). Late effects were frequent (59.7%), but prevalence rates differed across the diagnostic groups from 14.7% among MM survivors to 80.5% among BC survivors. BC survivors also reported the highest levels of fatigue and depression symptom severity compared with other diagnostic groups. Survivors of MM reported the least amount of somatic co-morbidity (53.6%), pain (7.8%), late effects (14.7%), anxiety, fatigue, depression, and PTSS. Survivors of BC and NHL had the highest PTSS scores (Table 1).

**Table 1:** Demographic characteristics of the sample in total and according to diagnostic group

	*N* (%)	MM^1^ (%)	BC^2^ (%)	CRC^3^ (%)	NHL^4^ (%)	LEU^5^ (%)
Total	936 (100)	231 (24.7)	359 (38.4)	110 (11.8)	146 (15.6)	90 (9.6)
Gender						
Female	682 (72.9)	163 (70.6)	359 (100)	55 (50)	66 (45.2)	39 (43.3)
Male	254 (27.1)	68 (29.4)	0 (0)	55 (50)	80 (54.8)	51 (56.7)
Education > 13 years	547 (58.4)	146 (63.2)	204 (56.8)	70 (63.6)	82 (56.2)	45 (50)
Not living alone	820 (82.2)	206 (89.2)	316 (88)	93 (84.5)	131 (84.5)	74 (82.2)
Living with children	446 (47.6)	119 (51.5)	170 (47.4)	44 (40)	68 (46.6)	45 (50)
Clinical variables						
Treatment modality						
Limited surgery^6^	216 (23.1)	216 (93.5)	0 (0)	0 (0)	0 (0)	0 (0)
Local treatment: surgery and/or RT^7^	106 (11.3)	0 (0)	30 (8.4)	69 (62.7)	7 (4.8)	0 (0)
Systemic treatment alone	108 (11.5)	9 (3.9)	2 (0.6)	2 (1.8)	36 (24.7)	59 (65.6)
Systemic treatments + surgery and/or RT	506 (54.1)	6 (2.6)	327 (91.1)	39 (35.5)	103 (70.5)	31 (34.4)
Somatic co-morbidity						
1–2 co-morbid somatic conditions	482 (51.5)	482 (50.2)	185 (51.5)	61 (55.5)	74 (50.7)	46 (51.1)
> 2 co-morbid somatic conditions	179 (19.1)	31 (13.4)	67 (18.7)	21 (19.1)	41 (28.1)	19 (21.1)
Pain interfering with normal work	93 (9.9)	18 (7.8)	39 (10.9)	11 (10.0)	16 (11.0)	9 (10.0)
Trouble sleeping	429 (45.8)	87 (37.7)	193 (53.8)	54 (49.1)	67 (45.9)	28 (31.1)
Late effects	559 (59.7)	34 (14.7)	289 (80.5)	56 (50.9)	112 (76.7)	68 (75.6)
Age at survey, mean (SD)^6^	49.1 (7.8)	49.4 (7.9)	49.8 (6.9)	49.1(9.4)	48.3 (8.2)	46.4(7.7)
Age at diagnosis, mean (SD)	32.6 (5.5)	31.2 (5.8)	35.2 (3.6)	33.5 (33.5)	30.3 (5.7)	28.8 (6)
Time since diagnosis, mean (SD)	15.9 (6.6)	17.7 (6.6)	14.0 (5.9)	15.1 (7.4)	17.4 (6.8)	16.9 (5.9)
HADS-A, mean (SD)	4.6 (3.7)	4.0 (3.3)	4.9 (3.8)	4.2 (3.4)	5.0 (16.4)	4.6 (3.7)
Fatigue, mean (SD)	12.9 (4.9)	12.1 (4.2)	13.5 (4.9)	13.0 (5.7)	13.1 (4.8)	12.4 (5.3)
PHQ, mean (SD)	5.1 (4.7)	3.8 (4)	5.8 (4.8)	5.1 (4.5)	5.5 (4.6)	5.0 (5.3)
PTSS, mean (SD)	11.8 (5.2)	10.0 (4.2)	12.7 (5.3)	11.7 (5.4)	12.7 (5.4)	11.6 (5.1)

^1^Malignant melanoma

^2^Breast cancer

^3^Colorectal cancer

^4^Non-hodgkin lymphoma

^5^Leukemia

^6^Standard deviation; MM survivors

^7^Radiation therapy

In the total sample, survivors worried most about getting another cancer (74%), followed by disease recurrence (69%), and then about future diagnostic tests (51%). Twenty percent reported quite a bit or a lot of worry concerning cancer recurrence and getting another cancer. The mean FCR score for the sample in total was 5.6, of whom survivors of BC had the highest (5.9), survivors of MM the second highest (5.6), and survivors of LEU the lowest (4.8) FCR score (Table 2).

**Table 2:** Fear of cancer recurrence (FCR) in the total sample, according to diagnostic group and cancer worry item in the Assessment of Survivorship Concern (ASC) Scale, and as a summed score, in total and according to diagnostic group

ASC item	*N* (%)	MM^1^ (%)	BC^2^ (%)	CRC^3^ (%)	NHL^4^ (%)	LEU^5^ (%)
1. I worry about future diagnostic tests						
Not at all	456 (48.9)	121 (52.8)	146 (40.8)	66 (60)	68 (46.6)	55 (61.1)
A little	391 (41.9)	98 (42.8)	170 (47.5)	34 (30.9)	60 (41.1)	29 (32.2)
Quite a bit	66 (7.1)	7 (3.1)	30 (8.4)	9 (8.2)	15 (10.3)	5 (5.6)
A lot	20 (2.1)	3 (1.3)	12 (3.4)	1 (0.9)	3 (2.1)	1 (1.1)
Total^6^	933	229	358	110	146	90
2. I worry about getting another cancer						
Not at all	243 (26)	47 (20.3)	87 (24.2)	39 (35.5)	36 (24.7)	34 (37.8)
A little	504 (53.8)	135 (58.4)	188 (52.4)	53 (48.2)	88 (60.3)	40 (44.4)
Quite a bit	148 (15.8)	38 (16.5)	67 (18.7)	12 (10.9)	20 (13.7)	11 (12.2)
A lot	41 (4.4)	11 (4.8)	17 (4.7)	6 (5.5)	2 (1.4)	5 (5.6)
Total	936	231	359	110	146	90
3. I worry about recurrence						
Not at all	288 (30.8)	56 (24.2)	76 (21.2)	42 (38.2)	56 (38.6)	58 (64.4)
A little	464 (49.6)	133 (57.6)	195 (54.3)	48 (43.6)	65 (44.8)	23 (25.6)
Quite a bit	135 (14.4)	32 (13.9)	65 (18.1)	11 (10)	21 (14.5)	6 (6.7)
A lot	48 (5.1)	10 (4.3)	23 (6.4)	9 (8.2)	3 (2.1)	3 (3.3)
Total^7^	935	231	359	110	145	90
FCR sum score, mean, (SD)^8^, (95% CI)^9^	5.55 (1.96) (5.42; 5.67)	5.56 (1.85) (5.32; 5.8)	5.87 (2.02) (5.66; 6.08)	5.25 (2.04) (4.86; 5.63)	5.39 (1.82) (5.09; 5.69)	4.81 (1.88) (4.42; 5.2)

^1^Malignant melanoma

^2^Breast cancer

^3^Colorectal cancer

^4^Non-hodgkin lymphoma

^5^Leukemia

^6^3 missing values for this item

^7^1 missing value for this item

^8^Standard deviation

^9^Confidence interval

Variables significantly associated with higher FCR score in the univariate linear regression models were being female, living with children, younger age at time of survey, and shorter time since diagnosis (Table 3). Undergoing local treatment (surgery and/or radiotherapy alone) was associated with lower risk of FCR compared with limited surgery (primarily MM survivors). Survivors of BC had increased risk of FCR compared with survivors of MM. More self-reported late effects and increased severity of fatigue, pain, trouble sleeping and depression, anxiety, and PTSS were all significantly associated with higher FCR, with the strongest associations observed for anxiety and PTSS (Table 3).

**Table 3:** Univariate linear regression analysis with FCR as the dependent variable

	Unstd. B (95% CI)^1^	Std β	*p* value	*R*^2^
Gender^2^	− 0.54 (− 0.82; − 0.26)	− 0.12	0.00	0.02
Age at survey	− 0.03 (− 0.04; − 0.01)	− 0.11	0.00	0.01
Education. > 13 years	0.19 (− 0.07; 0.44)	0.05	0.16	0.00
Living alone	− 0.23 (− 0.61; 0.16)	− 0.04	0.24	0.00
Living with children	0.42 (0.17; 0.67)	0.11	0.00	0.01
Diagnostic group^3^				0.03
BC	0.39 (0.19; 0.6)	0.17	0.00	
CRC	0.07 (− 0.13; 0.28)	0.03	0.48	
NHL	0.15 (− 0.06; 0.36)	0.06	0.15	
LEU	− 0.14 (− 0.35; 0.06)	− 0.05	0.18	
Time since diagnosis	− 0.04 (− 0.06; − 0.02)	− 0.14	0.00	0.02
Age at first diagnosis	0 (− 0.02; 0.03)	0.01	0.87	0.00
Treatment^4^				0.01
Local treatment	− 0.47 (− 0.93; − 0.02)	− 0.08	0.04	
Systemic treatment alone	− 0.26 (− 0.71; 0.19)	− 0.04	0.26	
Systemic treatments + surgery and/or RT	0.13 (− 0.18; 0.44)	0.03	0.41	
Somatic co-morbidity				0.00
1–2 co-morbid somatic conditions	0.24 (− 0.05; 0.54)	0.06	0.10	
> 2 co-morbid somatic conditions	0.12 (− 0.25; 0.49)	0.02	0.52	
Pain interfering with normal work	0.99 (0.57; 1.4)	0.15	0.00	0.02
Trouble sleeping	0.7 (0.45; 0.95)	0.18	0.00	0.03
Late effects	0.65 (0.4; 0.9)	0.16	0.00	0.03
Anxiety	0.24 (0.21; 0.27)	0.45	0.00	0.20
Fatigue	0.11 (0.08; 0.13)	0.26	0.00	0.07
Depression	0.15 (0.12; 0.17)	0.35	0.00	0.13
PTSS	0.24 (0.22; 0.25)	0.62	0.00	0.38

^1^Confidence interval

^2^Ref.: female

^3^Ref.: MM

^4^Ref.: limited surgery

In the first block of sociodemographic variables, being female, living with children, and younger age at survey were significantly associated with higher FCR and accounted for 3% (*R*^2^_adj_ 0.03, *p* < 0.01) of the variance in FCR scores. In block 2, none of the clinical variables included were significantly associated with FCR when adjusting for the effect of sociodemographic variables, explaining only an additional 2% (*R*^2^_adj_ 0.05, *p* < 0.01) of the variance in FCR scores. In block 3, entering health variables explained an additional 7% (*R*^*2*^_*adj*_ 0.12, p < 0.01) of the variance; living with children, trouble sleeping, and fatigue remained significantly associated with higher FCR. In block 4, entering late effects explained an additional 1% (*R*^2^_adj_ 0.13, *p* < 0.01) of the variance in FCR scores; living with children, trouble sleeping, late effects, and fatigue remained significantly associated with FCR. In block 5, entering anxiety resulted in a 12% (*R*^2^_adj_ 0.25, *p* < 0.01) additional explained variance in FCR and anxiety, late effects, living with children, and a previous BC diagnosis remained positively associated with FCR. In this block, BC was also associated with increased risk of FCR compared with MM (Std B 0.22, *p*: 0.04). In the final block entering PTSS, an additional 18% (*R*^2^ adj.0.43, *p* < 0.01) of the variance in the FCR score was explained, which was above all other variables in the model. PTSS was by far the strongest predictor of high FCR scores (Std B 0.21, *p* < 0.01), followed by being diagnosed with BC and CRC when compared with MM (Std B 0.31, *p* < 0.01 and std. B 0.55, *p*: 0.01, respectively), higher levels of anxiety (Std B 0.14, *p* < 0.01), and living with children (Std B 0.07, *p*: 0.01) (Table 4).

**Table 4:** Multivariate, hierarchical linear regression analysis with FCR as the dependent variable. Significant associations presented in italics

	Block 1			Block 2			Block 3			Block 4			Block 5			Block 6		
	*B* (95% CI)	Std. beta	*p*^*1*^	*B* (95% CI)^2^	Std. beta	*p*	*B* (95% CI)	Std. beta	*p*	*B* (95% CI)	Std. beta	*p*	*B* (95% CI)	Std. beta	*p*	*B* (95% CI)	Std beta	*p*
Constant	7.2 (6.21; 8.18)		< 0.01	6.87 (4.51; 9.22)		< 0.01	4.89 (2.57; 7.21)		< 0.01	4.44 (2.11; 6.77)		< 0.01	3.68 (1.51; 5.84)		< 0.01	1.57 (− 0.32; 3.47)		0.1
Gender^3^	*− 0.54 (− 0.82; − 0.26)*	*− 0.12*	*< 0.01*	− 0.24 (− 0.57; 0.09)	− 0.05	0.15	− 0.11 (− 0.43; 0.2)	− 0.03	0.479	− 0.09 (− 0.41; 0.22)	− 0.02	0.569	− 0.13 (− 0.42; 0.17)	− 0.03	0.39	− 0.02 (− 0.27; 0.24)	0.00	0.89
Living with children	*0.27 (0.01; 0.54)*	*0.07*	*0.05*	0.25 (− 0.02; 0.51)	0.06	0.067	*0.31 (0.05; 0.56)*	*0.08*	*0.019*	*0.31 (0.06; 0.57)*	*0.08*	*0.017*	*0.27 (0.03; 0.51)*	*0.07*	*0.025*	*0.28 (0.07; 0.48)*	*0.07*	*0.01*
Age at survey	*− 0.02 (− 0.04; − 0.01)*	*− 0.09*	*0.01*	− 0.02 (− 0.04; 0.01)	− 0.07	0.15	− 0.02 (− 0.04; 0.01)	− 0.06	0.208	− 0.02 (− 0.04; 0.01)	− 0.07	0.185	0 (− 0.03; 0.02)	− 0.02	0.728	− 0.01 (− 0.03; 0.01)	− 0.02	0.58
Time since diagnosis				− 0.01 (− 0.04; 0.02)	− 0.03	0.512	− 0.01 (− 0.04; 0.02)	− 0.04	0.467	− 0.01 (− 0.04; 0.02)	− 0.02	0.647	− 0.01 (− 0.04; 0.01)	− 0.04	0.357	0 (− 0.02; 0.02)	0.00	0.95
Diagnostic group^4^																		
BC				0.24 (− 0.28; 0.77)	0.11	0.366	0.36 (− 0.14; 0.87)	0.16	0.161	0.47 (− 0.04; 0.98)	0.21	0.07	*0.5 (0.03; 0.97)*	*0.22*	*0.038*	*0.69 (0.28; 1.1)*	*0.31*	*< 0.01*
CRC				0.11 (− 0.41; 0.63)	0.04	0.674	0.19 (− 0.31; 0.69)	0.07	0.458	0.32 (− 0.19; 0.82)	0.11	0.215	0.46 (− 0.01; 0.92)	0.16	0.054	*0.55 (0.14; 0.95)*	*0.19*	*0.01*
NHL				0.02 (− 0.51; 0.55)	0.01	0.947	0.14 (− 0.38; 0.65)	0.05	0.602	0.23 (− 0.28; 0.75)	0.09	0.375	0.26 (− 0.22; 0.74)	0.10	0.286	0.4 (− 0.01; 0.82)	0.15	0.06
LEU				− 0.38 (− 0.94; 0.18)	− 0.13	0.186	− 0.2 (− 0.75; 0.34)	− 0.07	0.467	− 0.12 (− 0.66; 0.42)	− 0.04	0.664	− 0.07 (− 0.57; 0.44)	− 0.02	0.801	0.17 (− 0.27; 0.61)	0.06	0.44
Treatment^5^																		
Local treatment				− 0.69 (− 1.84; 0.47)	− 0.11	0.243	− 0.43 (− 1.55; 0.68)	− 0.07	0.444	− 0.39 (− 1.5; 0.72)	− 0.06	0.49	− 0.41 (− 1.43; 0.62)	− 0.07	0.438	− 0.01 (− 0.9; 0.89)	0.00	0.99
Systemic treatment alone				0.12 (− 0.91; 1.14)	0.02	0.825	0.24 (− 0.75; 1.23)	0.04	0.633	0.2 (− 0.79; 1.18)	0.03	0.695	0.28 (− 0.63; 1.19)	0.05	0.546	0.33 (− 0.46; 1.13)	0.05	0.41
Systemic treatments + RT and/or surgery				− 0.24 (− 1.29; 0.82)	− 0.06	0.66	− 0.15 (− 1.16; 0.87)	− 0.04	0.78	− 0.26 (− 1.27; 0.75)	− 0.07	0.61	− 0.09 (− 1.03; 0.85)	− 0.02	0.855	0.03 (− 0.79; 0.84)	0.01	0.95
Pain							0.33 (− 0.11; 0.76)	0.05	0.143	0.28 (− 0.15; 0.72)	0.04	0.2	0.2 (− 0.21; 0.6)	0.03	0.335	− 0.18 (− 0.54; 0.17)	− 0.03	0.31
Trouble sleeping							*0.41 (0.16; 0.67)*	*0.11*	*< 0.01*	*0.37 (0.12; 0.63)*	*0.10*	*< 0.01*	0.04 (− 0.2; 0.28)	0.01	0.75	0.02 (− 0.19; 0.23)	0.01	0.86
Fatigue							*0.08 (0.05; 0.11)*	*0.20*	*< 0.01*	*0.07 (0.05; 0.1)*	*0.18*	*< 0.01*	0.02 (− 0.01; 0.05)	0.05	0.156	− 0.01 (− 0.03; 0.01)	− 0.03	0.41
Late effects										*0.51 (0.2; 0.82)*	*0.13*	*< 0.01*	*0.44 (0.16; 0.73)*	*0.11*	*< 0.01*	0.18 (− 0.07; 0.43)	0.05	0.16
Anxiety													*0.21 (0.18; 0.25)*	*0.40*	*< 0.01*	*0.08 (0.04; 0.11)*	*0.14*	*< 0.01*
PTSS																*0.21 (0.19; 0.24)*	*0.56*	*< 0.01*
*R*^2^_adj_	0.03			0.05			0.12			0.13			0.25			0.43		

^1^*p* value

^2^Confidence interval

^3^Reference: female

^4^Reference: MM

^5^Reference: limited surgery

## Discussion

This study demonstrates that FCR is frequent even decades beyond treatment completion in a large population-based sample of young adult cancer survivors, representing a range of different cancer diagnoses, and also among survivors of cancers associated with a favorable prognosis, such as MM. Between 69 and 75% reported some degree of FCR and approximately 20% reported quite a bit or a lot of worry concerning disease recurrence or getting another cancer in this large population of long-term survivors. Previous studies on FCR among AYA cancer survivors, which typically have shorter observation times, report prevalence estimates in the range of 31–85% [7, 11]. The heterogeneity of both samples and FCR measures used makes comparison across studies challenging. Our finding is however in keeping with the abovementioned paper by Skaali et al. on FCR among long-term testicular cancer survivors in Norway, a cohort of survivors comparable with ours in regard to age at diagnosis and follow-up time [21].

Living with children was the only sociodemographic variable demonstrating a consistent increased risk of FCR. This factor seems more important for FCR risk than age, educational level, and partner support in our sample. This has been reported in previous single studies; Mehnert et al. explored FCR among BC survivors, and reported a significant association between FCR and having children [35], but the association has not been consistent. In the review by Yang et al., two studies explored the effect of having children on FCR risk and reported a nil association [7]. Cancer is an existential threat, not only for the patient but also for the family as a whole. It is therefore reasonable to deduce that being aware that their cancer may impact their children emotionally, financially, and practically may increase survivors’ FCR. Female gender was associated with higher FCR in the univariate model. The highest FCR levels were found among BC and MM survivors, which were the diagnostic groups with the highest proportion of females. The effect of gender was, however, attenuated when entering clinical variables in the multivariable models suggesting that other factors are important for understanding FCR in long-term young adult cancer survivors.

Women with BC report the most cancer worry and highest FCR score in this sample. This is concurrent with similar findings among older cancer survivors. In fact, FCR has been described as a crucial long-term, unmet supportive care need among adult BC survivors [36]. Young adults with BC have, in general, more aggressive tumor characteristics compared with patients > 40 years, including more advanced disease, triple-negative, and HER-2 positive tumors [37], making BC the leading cause of cancer-related death in this age group. Young BC survivors are likely aware that their low age at diagnosis is a negative prognostic factor. Furthermore, these survivors reported a high degree of late effects, including pain, which may serve as constant reminders of their cancer illness, and also can be misinterpreted as signs of relapse.

Survivors of MM reported the second highest FCR scores in this population, despite the majority having undergone minimal treatment, and are considered to have excellent long-term prognosis. These survivors had the least amount of somatic co-morbidities and psychological co-morbidity scores of the sample. A high degree of FCR among MM survivors may be due to patient awareness of the fact that metastatic MM has dismal prognosis [38]. Furthermore, there is a high degree of public focus on individual prevention of MM through self-checking and self-awareness through population health campaigns and in the mass media. As a result, survivors of MM may feel especially at risk of recurrence compared with other diagnostic groups not so frequently presented in mass media. Also, patients treated for localized MM may not receive oncological or other follow-up care as many are treated by dermatologists, surgeons, or general practitioners rather than oncologists. In a meta-analysis by Tauber et al., exploring the effect of psychological interventions on FCR risk, the authors concluded with a moderate but robust effect of cognitive behavioral therapies (CBT) on symptom intensity [39], which could indicate a beneficial effect of oncological follow-up care. The opposite may, however, also be the case. Among BC survivors having mammograms, McGinty et al., reported that FCR increased in intensity prior to the screening exam, before declining again when the results came back negative [40]. That FCR is present also among survivors with good prognosis indicate that subjective perception of recurrence risk is of importance. This is in line with the CBM of health anxiety [41], which propose that regular checkups with an oncologist, which is meant to provide reassurance, may in fact increase cognitions about recurrence risk and subsequently symptom intensity.

FCR was positively associated with anxiety and PTSS, and the multivariate analyses clearly demonstrate that PTSS has the strongest association with FCR. A cancer diagnosis is stressful, and is sufficiently traumatic to induce PTSD, albeit in a minority of patients [42]. Younger age has been reported to be a risk factor for this to occur. PTSS, on the other hand, seems much more prevalent also among young adults after cancer, for instance in a study by McCarthy et al., where PTSS was present in almost half of the examined population (15–25 years) [14]. A study by Smith et al. concluded that PTSS predicted FCR morbidity in adult patients with BC, CRC, and MM [20]. In line with our findings, they found that demographic and clinical characteristics have a weaker association to FCR compared with psychological variables. Skaali et al. reported that increasing levels of traumatic cancer-related stress symptoms was significantly associated with rising FCR [21].

It may be argued that FCR, anxiety, and PTSS are closely related with overlapping symptomatology. The inter-correlations between these measures were, however, only modest, suggesting that they tap distinct underlying constructs. The Impact of Events Scale-6 (IES-6) is used to measure cancer-related PTSS and map out intrusive thoughts and avoidance behavior during the last week, and is widely used to assess the impact of cancer-specific distress. The Assessment of Survivor Concern (ASC) Scale, used to identify FCR in our study, is developed to identify cancer-related worries specifically. Both the IES-6 and the ASC are reported to have a high degree of validity [25, 34]. In this study, PTSS was added to the multivariable model after controlling for the effect of anxiety. Although adding PTSS to the model weakened the effect of anxiety on FCR, it remained moderately associated with FCR. This suggests that PTSS could potentially mediate the effect of anxiety on FCR, although the cross-sectional nature of our sample does not allow for such an investigation.

We expected that physical and psychological late effects could serve as “cancer-cues” triggering FCR. Pain, trouble sleeping, fatigue, or other late effects were significantly associated with FCR in unadjusted analyses, and also in the multivariate model until adjustment for PTSS were made. This may suggest that late effects impact FCR indirectly via PTSS. To the best of our knowledge, a link between late effects, PTSS and FCR has not been previously reported among young adult cancer survivors across diagnostic groups, but is in line with previous findings among survivors of childhood cancer [22], where pain and fatigue were associated with higher risk of FCR.

The need for age-sensitive and comprehensive follow-up care of cancer survivors will become increasingly important as cancer survival rates continue to improve, resulting in a growing population of long-term survivors. As evidence of the relative high prevalence of FCR, its distress and impact on quality of life is gaining momentum, also in the AYA cancer survivor population—and there are efficacious therapies available; the clinical community should, in our view, put forward a stronger focus on FCR in follow-up care, which includes referral to psycho-oncological services when indicated. Whether all survivors should be screened for FCR, or if it should be reserved for at-risk subsets of survivors, needs to be further discussed. This is further dependent upon reaching a consensus on how to best measure FCR. Such an agreement will simplify future research focusing on finding FCR management strategies suitable for implementation in routine clinical practices [43].

### Strengths and limitation

The current study is nation-wide and population-based, representing a large unselected cohort of survivors identified by the CRN, which is of high quality, based on mandatory reporting and near-to-complete for the entire population [26]. This has enabled the inclusion of a high number of long-term cancer survivors not otherwise engaged in follow-up care, providing data with high internal and external validity. Although the NOR-CAYACS study had a modest response rate of 42%, there is little evidence to suggest a non-response bias in the data [24]. We cannot, however, exclude the possibility that such bias exists for the examined associations. The most prevalent cancer diagnoses among AYA survivors were included in this study with the exception of testicular and cervical cancer. This was due to concurrent studies at the time of study inclusion, resulting in a primarily female study population. Although we adjusted for the effect of gender in the multivariate analyses, we cannot rule out the risk of potential selection bias. The inclusion of male AYA cancer survivors will be important in future cancer survivorship research projects. In general, the cross-sectional design does not allow for causal inference. Therefore, we cannot know the causal relationships between anxiety, PTSS, and FCR, which should be the subject for future longitudinal, prospective studies.

How to measure and capture clinically significant FCR has been subject to considerable debate. A systematic review of self-reported measures for FCR identified 20 different tools [44]. This is a reflection of the heterogeneity of FCR definitions that have been applied. The consensus definition of FCR was introduced in 2016 [5], a year later than the design of the NOR-CAYACS study. As a result, prevalence estimates of FCR in previous research vary greatly, and pooled estimates have a wide range, as demonstrated in the review by Yang et al. [7]. The ASC is a brief instrument, where only three items specifically target cancer worry, and it does not separate between clinically significant versus lower levels of concern. It is at the discretion of the clinician to decide appropriate cutoff levels (written correspondence with the author). To our knowledge, the ASC has not been validated specifically in this age group, nor does it explore the potential impact of the condition, and no data on stability or sensitivity of the ASC are available [44]. The ASC has however reported excellent validity and internal consistency, and is, despite the abovementioned limitations, recommended for assessing cancer survivor worry [25].

## Conclusions

FCR is a frequent concern among survivors of young adult cancers more than 15 years after diagnosis. The strongest predictor of FCR was PTSS, above and beyond any demographic or clinical variable. Survivors of BC and MM report higher levels of FCR than LEU, CRC, and NHL. This is the first study to report this late effect among young MM survivors. The clinical implication may be substantial as this increasing group of survivors is likely to have unmet supportive follow-up needs. Clinical awareness of FCR, also when risk of cancer recurrence is low, is warranted, not only from the oncological community but also from other health professionals caring for cancer survivors. It is imperative that the recently reached consensus on what constitutes clinical FCR translate into future research, and that a similar consensus is reached concerning what instrument to use, in order to develop standardized clinical guidelines concerning the identification and treatment of FCR.

## Data Availability

Data included in this paper was provided by the Norwegian Cancer Registry and collected through the NOR-CAYACS survey. All data are available at the National advisory unit of late effects after cancer treatment, Oslo University Hospital, Oslo, Norway.

## References

[1] Lewis DR, Seibel NL, Smith AW, Stedman MR (2014). Adolescent and young adult cancer survival. J Natl Cancer Inst Monogr.

[2] Barr RD, Ferrari A, Ries L, Whelan J, Bleyer WA (2016). Cancer in adolescents and young adults: a narrative review of the current status and a view of the future. JAMA Pediatr.

[3] Smith AW, Bellizzi KM, Keegan TH, Zebrack B, Chen VW, Neale AV, Hamilton AS, Shnorhavorian M, Lynch CF (2013). Health-related quality of life of adolescent and young adult patients with cancer in the United States: the adolescent and young adult health outcomes and patient experience study. Journal of clinical oncology : official journal of the American Society of Clinical Oncology.

[4] Mertens AC, Yasui Y, Neglia JP, Potter JD, Nesbit ME, Ruccione K, Smithson WA, Robison LL (2001). Late mortality experience in five-year survivors of childhood and adolescent cancer: the childhood cancer survivor study. Journal of clinical oncology : official journal of the American Society of Clinical Oncology.

[5] Lebel S, Ozakinci G, Humphris G, Mutsaers B, Thewes B, Prins J, Dinkel A, Butow P (2016). From normal response to clinical problem: definition and clinical features of fear of cancer recurrence. Supportive care in cancer : official journal of the Multinational Association of Supportive Care in Cancer.

[6] Simard S, Thewes B, Humphris G, Dixon M, Hayden C, Mireskandari S, Ozakinci G (2013). Fear of cancer recurrence in adult cancer survivors: a systematic review of quantitative studies. Journal of cancer survivorship : research and practice.

[7] Yang Y, Li W, Wen Y, Wang H, Sun H, Liang W, Zhang B, Humphris G (2019). Fear of cancer recurrence in adolescent and young adult cancer survivors: a systematic review of the literature. Psycho-oncology.

[8] Sender A, Friedrich M, Leuteritz K, Nowe E, Stöbel-Richter Y, Mehnert A, Geue K (2019). Unmet supportive care needs in young adult cancer patients: associations and changes over time. Results from the AYA-Leipzig study. Journal of cancer survivorship : research and practice.

[9] Gittzus JA, Fasciano KM, Block SD, Mack JW. Peace of mind among adolescents and young adults with cancer. Psycho-oncology. 2019. 10.1002/pon.5309 31825157

[10] Society for Adolescent H (2017). Medicine: young adult health and well-being: a position statement of the Society for Adolescent Health and Medicine. J Adolesc Health.

[11] Thewes B, Kaal SEJ, Custers JAE, Manten-Horst E, Jansen R, Servaes P, van der Graaf WTA, Prins JB, Husson O (2018). Prevalence and correlates of high fear of cancer recurrence in late adolescents and young adults consulting a specialist adolescent and young adult (AYA) cancer service. Supportive care in cancer : official journal of the Multinational Association of Supportive Care in Cancer.

[12] Seitz DC, Besier T, Debatin KM, Grabow D, Dieluweit U, Hinz A, Kaatsch P, Goldbeck L (2010). Posttraumatic stress, depression and anxiety among adult long-term survivors of cancer in adolescence. Eur J Cancer (Oxford, England : 1990).

[13] Allen J, Willard VW, Klosky JL, Li C, Srivastava DK, Robison LL, Hudson MM, Phipps S (2018). Posttraumatic stress-related psychological functioning in adult survivors of childhood cancer. Journal of cancer survivorship : research and practice.

[14] McCarthy MC, McNeil R, Drew S, Dunt D, Kosola S, Orme L, Sawyer SM (2016). Psychological distress and posttraumatic stress symptoms in adolescents and young adults with cancer and their parents. Journal of adolescent and young adult oncology.

[15] Kwak M, Zebrack BJ, Meeske KA, Embry L, Aguilar C, Block R, Hayes-Lattin B, Li Y, Butler M, Cole S (2013). Prevalence and predictors of post-traumatic stress symptoms in adolescent and young adult cancer survivors: a 1-year follow-up study. Psycho-oncology.

[16] Lee-Jones C, Humphris G, Dixon R, Hatcher MB (1997). Fear of cancer recurrence--a literature review and proposed cognitive formulation to explain exacerbation of recurrence fears. Psycho-oncology.

[17] Mutsaers B, Butow P, Dinkel A, Humphris G, Maheu C, Ozakinci G, Prins J, Sharpe L, Smith AB, Thewes B (2020). Identifying the key characteristics of clinical fear of cancer recurrence: an international Delphi study. Psycho-oncology.

[18] Custers JA, Gielissen MF, de Wilt JH, Honkoop A, Smilde TJ, van Spronsen DJ, van der Veld W, van der Graaf WT, Prins JB (2017). Towards an evidence-based model of fear of cancer recurrence for breast cancer survivors. Journal of cancer survivorship : research and practice.

[19] Ozga M, Aghajanian C, Myers-Virtue S, McDonnell G, Jhanwar S, Hichenberg S, Sulimanoff I (2015). A systematic review of ovarian cancer and fear of recurrence. Palliative & supportive care.

[20] Smith A, Sharpe L, Thewes B, Turner J, Gilchrist J, Fardell JE, Girgis A, Tesson S, Descallar J, Bell ML (2018). Medical, demographic and psychological correlates of fear of cancer recurrence (FCR) morbidity in breast, colorectal and melanoma cancer survivors with probable clinically significant FCR seeking psychological treatment through the ConquerFear study. Supportive care in cancer : official journal of the Multinational Association of Supportive Care in Cancer.

[21] Skaali T, Fosså SD, Bremnes R, Dahl O, Haaland CF, Hauge ER, Klepp O, Oldenburg J, Wist E, Dahl AA (2009). Fear of recurrence in long-term testicular cancer survivors. Psycho-oncology.

[22] Kelada L, Wakefield CE, Heathcote LC, Jaaniste T, Signorelli C, Fardell JE, Donoghoe M, McCarthy MC, Gabriel M, Cohn RJ, ANZCHOG survivorship study group (2019). Perceived cancer-related pain and fatigue, information needs, and fear of cancer recurrence among adult survivors of childhood cancer. Patient Educ Couns.

[23] Cho D, Chu Q, Lu Q (2018). Associations among physical symptoms, fear of cancer recurrence, and emotional well-being among Chinese American breast cancer survivors: a path model. Supportive care in cancer : official journal of the Multinational Association of Supportive Care in Cancer.

[24] Lie HC, Rueegg CS, Fossa SD, Loge JH, Ruud E, Kiserud CE (2019). Limited evidence of non-response bias despite modest response rate in a nationwide survey of long-term cancer survivors-results from the NOR-CAYACS study. Journal of cancer survivorship : research and practice.

[25] Gotay CC, Pagano IS (2007). Assessment of Survivor Concerns (ASC): a newly proposed brief questionnaire. Health Qual Life Outcomes.

[26] Larsen IK, Småstuen M, Johannesen TB, Langmark F, Parkin DM, Bray F, Møller B (2009). Data quality at the Cancer Registry of Norway: an overview of comparability, completeness, validity and timeliness. European journal of cancer (Oxford, England : 1990).

[27] Brusselaers N, Lagergren J (2017). The Charlson comorbidity index in registry-based research. Methods Inf Med.

[28] Bøhn S-KH, Thorsen L, Kiserud CE, Fosså SD, Lie HC, Loge JH, Wisløff T, Haugnes HS, Reinertsen KV (2019). Chronic fatigue and associated factors among long-term survivors of cancers in young adulthood. Acta oncologica (Stockholm, Sweden).

[29] Ware J, Kosinski M, Keller SD (1996). A 12-item short-form health survey: construction of scales and preliminary tests of reliability and validity. Med Care.

[30] Krokstad S, Langhammer A, Hveem K, Holmen TL, Midthjell K, Stene TR, Bratberg G, Heggland J, Holmen J (2013). Cohort profile: the HUNT study, Norway. Int J Epidemiol.

[31] Chalder T, Berelowitz G, Pawlikowska T, Watts L, Wessely S, Wright D, Wallace EP (1993). Development of a fatigue scale. J Psychosom Res.

[32] Kroenke K, Spitzer RL, Williams JB (2001). The PHQ-9: validity of a brief depression severity measure. J Gen Intern Med.

[33] Zigmond AS, Snaith RP (1983). The hospital anxiety and depression scale. Acta Psychiatr Scand.

[34] Thoresen S, Tambs K, Hussain A, Heir T, Johansen VA, Bisson JI (2010). Brief measure of posttraumatic stress reactions: Impact of Event Scale-6. Soc Psychiatry Psychiatr Epidemiol.

[35] Mehnert A, Berg P, Henrich G, Herschbach P (2009). Fear of cancer progression and cancer-related intrusive cognitions in breast cancer survivors. Psycho-oncology.

[36] Fang SY, Fetzer SJ, Lee KT, Kuo YL (2018). Fear of recurrence as a predictor of care needs for long-term breast cancer survivors. Cancer Nurs.

[37] Murphy BL, Day CN, Hoskin TL, Habermann EB, Boughey JC (2019). Adolescents and young adults with breast cancer have more aggressive disease and treatment than patients in their forties. Ann Surg Oncol.

[38] von Schuckmann LA, Hughes MCB, Ghiasvand R, Malt M, van der Pols JC, Beesley VL, Khosrotehrani K, Smithers BM, Green AC (2019). Risk of melanoma recurrence after diagnosis of a high-risk primary tumor. JAMA Dermatol.

[39] Tauber NM, O’Toole MS, Dinkel A, Galica J, Humphris G, Lebel S, Maheu C, Ozakinci G, Prins J, Sharpe L (2019). Effect of psychological intervention on fear of cancer recurrence: a systematic review and meta-analysis. Journal of clinical oncology : official journal of the American Society of Clinical Oncology.

[40] McGinty HL, Small BJ, Laronga C, Jacobsen PB (2016). Predictors and patterns of fear of cancer recurrence in breast cancer survivors. Health psychology : official journal of the Division of Health Psychology, American Psychological Association.

[41] Warwick HM, Salkovskis PM (1985). Reassurance. Br Med J (Clin Res Ed).

[42] Abbey G, Thompson SB, Hickish T, Heathcote D (2015). A meta-analysis of prevalence rates and moderating factors for cancer-related post-traumatic stress disorder. Psycho-oncology.

[43] Jacobsen PB (2017). New challenges in psycho-oncology research II: a health care delivery, dissemination, and implementation research model to promote psychosocial care in routine cancer care. Psycho-oncology.

[44] Thewes B, Butow P, Zachariae R, Christensen S, Simard S, Gotay C (2012). Fear of cancer recurrence: a systematic literature review of self-report measures. Psycho-oncology.

